# 2-[(4-Chloro­phen­yl)(2-hy­droxy-5-oxo­cyclo­pent-1-en-1-yl)meth­yl]-3-hy­droxy­cyclo­pent-2-en-1-one

**DOI:** 10.1107/S1600536812018715

**Published:** 2012-05-12

**Authors:** Gongzhen Li, Peijun Cai, Junhao Huo, Chong Shi

**Affiliations:** a School of Chemical Engineering and Technology, China University of Mining and Technology, Xuzhou Jiangsu 221116, People’s Republic of China; b School of Environment Science and Spatial Informatics, China University of Mining and Technology, Xuzhou Jiangsu 221116, People’s Republic of China

## Abstract

There are two mol­ecules in the asymmetric unit of the title compound, C_17_H_15_ClO_4_, in which the dihedral angles between the five-membered rings are 57.3 (1) and 51.4 (1)°. An intra­molecular O—H⋯O hydrogen bond occurs in each mol­ecule. In the crystal, O—H⋯O and C—H⋯O hydrogen bonds link the moleclues into chains along the *b* axis.

## Related literature

For the ability of carbon atoms to act as proton donors in hydrogen bonds, see: Allen *et al.* (1996 ▶); Sutor (1963 ▶); Venkatesan *et al.* (2004 ▶); Wang *et al.* (2005 ▶); Zhu *et al.* (2005 ▶).
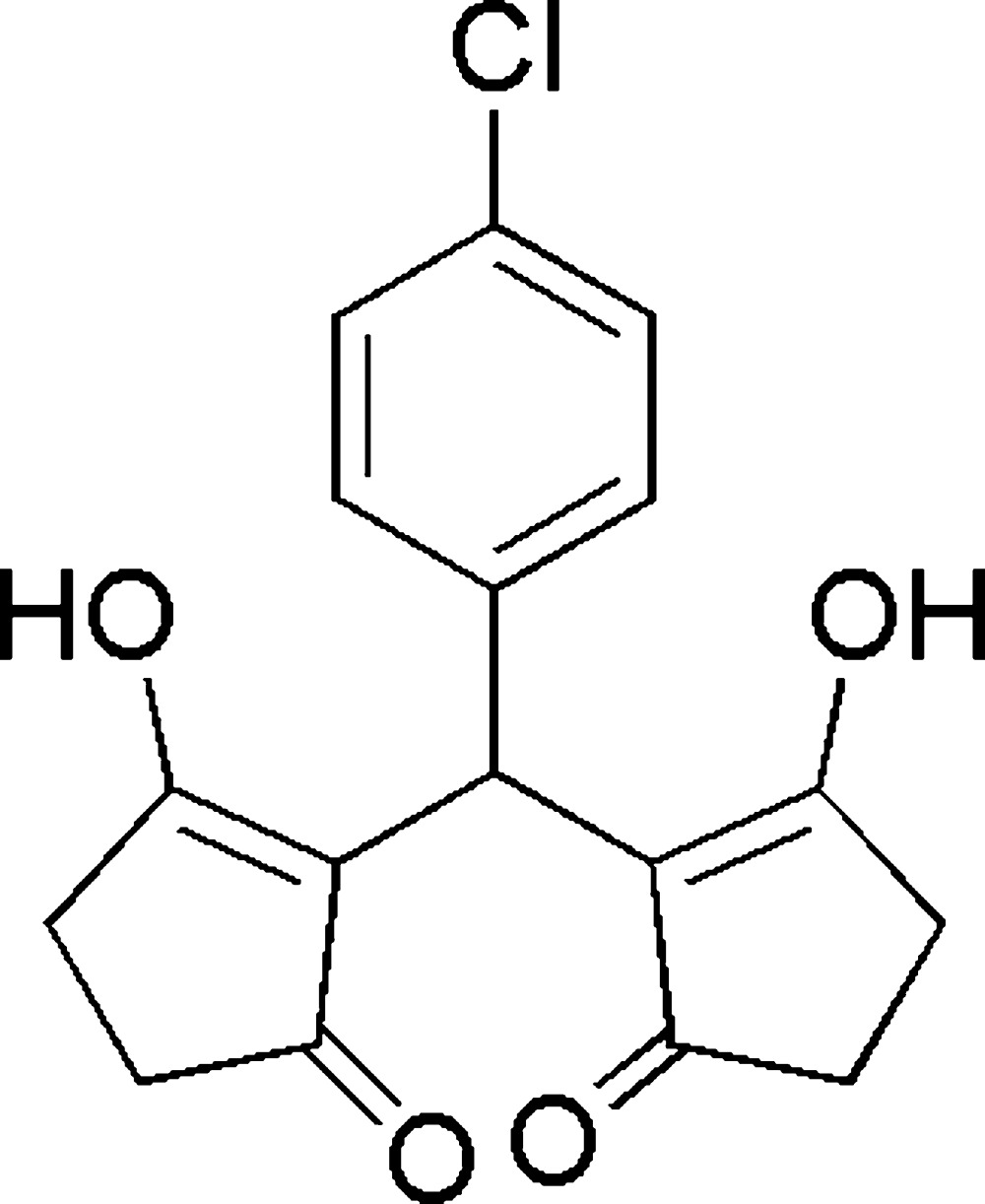


## Experimental

### 

#### Crystal data

C_17_H_15_ClO_4_
*M* *_r_* = 318.74
Monoclinic, 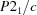

*a* = 14.177 (2) Å
*b* = 10.4002 (18) Å
*c* = 21.347 (4) Å
β = 100.621 (2)°
*V* = 3093.6 (9) Å^3^
*Z* = 8
Mo *K*α radiation
μ = 0.26 mm^−1^
*T* = 296 K
0.36 × 0.19 × 0.13 mm

#### Data collection

Bruker SMART APEXII CCD area-detector diffractometer
21681 measured reflections
5521 independent reflections
4756 reflections with *I* > 2σ(*I*)
*R*_int_ = 0.020

#### Refinement

*R*[*F* ^2^ > 2σ(*F* ^2^)] = 0.037
*wR*(*F* ^2^) = 0.105
*S* = 1.03
5521 reflections
414 parameters
4 restraints
H atoms treated by a mixture of independent and constrained refinement
Δρ_max_ = 0.35 e Å^−3^
Δρ_min_ = −0.36 e Å^−3^

### 

Data collection: *APEX2* (Bruker, 2004 ▶); cell refinement: *SAINT* (Bruker, 2004 ▶); data reduction: *SAINT*; program(s) used to solve structure: *SHELXTL* (Sheldrick, 2008 ▶); program(s) used to refine structure: *SHELXTL*; molecular graphics: *SHELXTL*; software used to prepare material for publication: *SHELXTL*.

## Supplementary Material

Crystal structure: contains datablock(s) global, I. DOI: 10.1107/S1600536812018715/wn2471sup1.cif

Structure factors: contains datablock(s) I. DOI: 10.1107/S1600536812018715/wn2471Isup2.hkl

Supplementary material file. DOI: 10.1107/S1600536812018715/wn2471Isup3.cml

Additional supplementary materials:  crystallographic information; 3D view; checkCIF report

## Figures and Tables

**Table 1:** Hydrogen-bond geometry (Å, °)

*D*—H⋯*A*	*D*—H	H⋯*A*	*D*⋯*A*	*D*—H⋯*A*
O5—H5⋯O7	0.87 (2)	1.66 (2)	2.529 (2)	171 (3)
O3—H3⋯O1	0.87 (2)	1.70 (2)	2.558 (2)	169 (3)
O2—H2⋯O4^i^	0.86 (2)	1.69 (2)	2.5424 (16)	173 (3)
O8—H8⋯O6^ii^	0.86 (2)	1.71 (2)	2.5564 (16)	171 (2)
C9—H9*A*⋯O5^iii^	0.97	2.52	3.277 (2)	134

Symmetry codes: (i) 
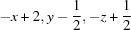
; (ii) 
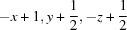
; (iii) 
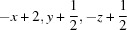
.

**Fig. 1.**
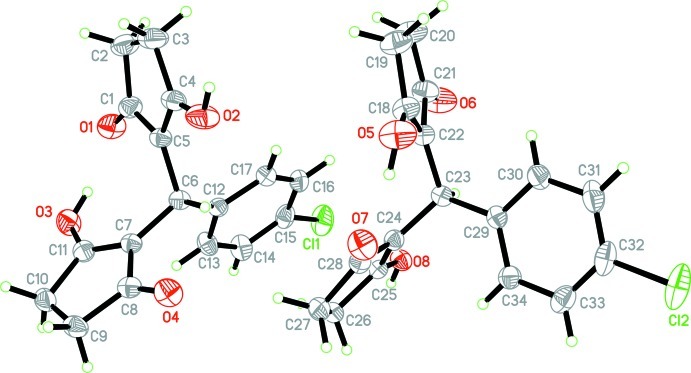
The structure of the asymmetric unit, showing 30% probability displacement ellipsoids. Hydrogen atoms are shown as spheres of arbitrary radius.

**Fig. 2.**
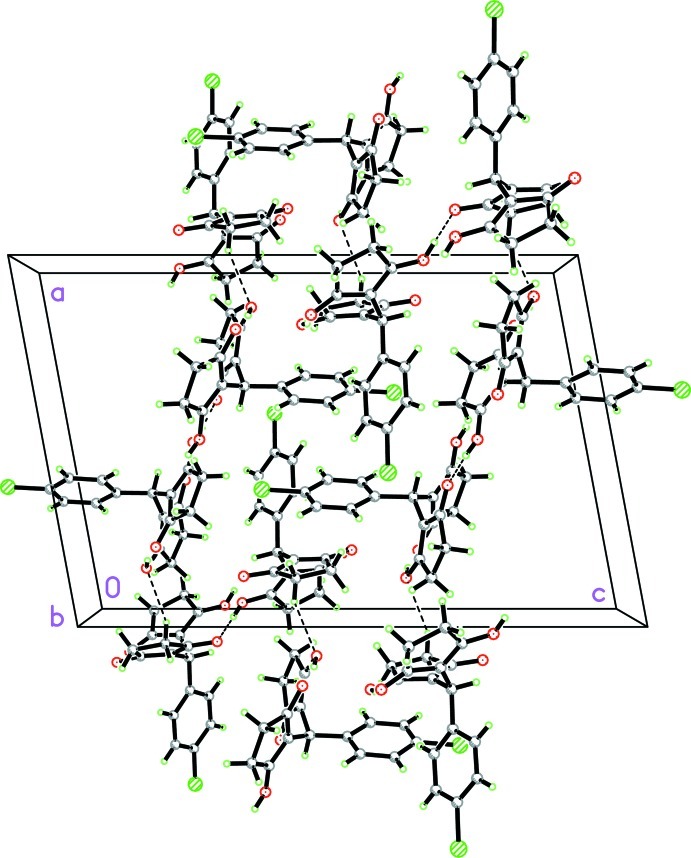
The packing diagram of the crystal structure. Dashed lines indicate hydrogen bonds.

## supplementary crystallographic information

### Comment

The ability of carbon atoms to act as proton donors in hydrogen bonds has been recognized for many years and reports of these non-classical hydrogen bonds have been reported in crystallographic studies (Allen *et al.*, 1996; Sutor, 1963; Zhu *et al.*, 2005; Wang *et al.*, 2005) and spectroscopic studies (Venkatesan *et al.*, 2004). We continue the study of such interactions with the crystal structure of the title compound.

In its crystal structure, there are two independent molecules in the asymmetric unit. All of the five-membered rings are planar. In molecule 1, the dihedral angles between the benzene ring and the two cyclopentene rings are 86.8 (1)° and 65.6 (1)°; the angle between the two five membered rings is 57.3 (1)°. In molecule 2, the dihedral angles between the benzene ring and the two cyclopentene rings are 73.1 (1)° and 80.0 (1)°; the angle between the two five membered rings is 51.4 (1)°.

The intermolecular hydrogen bonds O2—H2···O4, O8—H8···O6 and C9—H9A···O5 link adjacent moleclues, forming an infinite one-dimensional chain along the *b* axis. Furthermore, each molecule of the asymmetric unit exhibits an intramolecular O—H···O hydrogen bond.

### Experimental

The title compound was prepared by the reaction of 4-chlorobenzaldehyde (0.146 g, 1.0 mmol), cyclopentane-1,3-dione (0.196 g, 2.0 mmol), and liquid 1-butyl-3-methylimidazolium bromide (2.0 ml) for 8 h at 353 K (yield 76%, mp. 517–519 K). Crystals suitable for X-ray diffraction were obtained by slow evaporation of a dimethylformamide solution.

### Refinement

The oxygen-bound H atoms were positioned geometrically and refined to distance values in the range O—H = 0.856 (15) - 0.872 (16) Å. Carbon-bound H atoms were positioned geometrically and refined as riding, with C—H = 0.93–0.98 Å, and with *U*_iso_(H) = 1.2*U*_eq_(C).

### Crystal data

**Table:**

C_17_H_15_ClO_4_	*D*_x_ = 1.369 Mg m^−^^3^
*M**_r_* = 318.74	Melting point = 517–519 K
Monoclinic, *P*2_1_/*c*	Mo *K*α radiation, λ = 0.71073 Å
*a* = 14.177 (2) Å	Cell parameters from 9980 reflections
*b* = 10.4002 (18) Å	θ = 2.4–26.8°
*c* = 21.347 (4) Å	µ = 0.26 mm^−^^1^
β = 100.621 (2)°	*T* = 296 K
*V* = 3093.6 (9) Å^3^	Block, colourless
*Z* = 8	0.36 × 0.19 × 0.13 mm
*F*(000) = 1328	

### Data collection

**Table:**

Bruker SMART APEXII CCD area-detector diffractometer	4756 reflections with *I* > 2σ(*I*)
Radiation source: fine-focus sealed tube	*R*_int_ = 0.020
Graphite monochromator	θ_max_ = 25.2°, θ_min_ = 2.6°
phi and ω scans	*h* = −16→15
21681 measured reflections	*k* = −12→12
5521 independent reflections	*l* = −25→24

### Refinement

**Table:**

Refinement on *F*^2^	Secondary atom site location: difference Fourier map
Least-squares matrix: full	Hydrogen site location: inferred from neighbouring sites
*R*[*F*^2^ > 2σ(*F*^2^)] = 0.037	H atoms treated by a mixture of independent and constrained refinement
*wR*(*F*^2^) = 0.105	*w* = 1/[σ^2^(*F*_o_^2^) + (0.0562*P*)^2^ + 0.8138*P*] where *P* = (*F*_o_^2^ + 2*F*_c_^2^)/3
*S* = 1.03	(Δ/σ)_max_ = 0.001
5521 reflections	Δρ_max_ = 0.35 e Å^−^^3^
414 parameters	Δρ_min_ = −0.36 e Å^−^^3^
4 restraints	Extinction correction: *SHELXTL* (Sheldrick, 2008), Fc^*^=kFc[1+0.001xFc^2^λ^3^/sin(2θ)]^-1/4^
Primary atom site location: structure-invariant direct methods	Extinction coefficient: 0.0128 (9)

### Special details

**Table:**

Geometry. All e.s.d.'s (except the e.s.d. in the dihedral angle between two l.s. planes) are estimated using the full covariance matrix. The cell e.s.d.'s are taken into account individually in the estimation of e.s.d.'s in distances, angles and torsion angles; correlations between e.s.d.'s in cell parameters are only used when they are defined by crystal symmetry. An approximate (isotropic) treatment of cell e.s.d.'s is used for estimating e.s.d.'s involving l.s. planes.
Refinement. Refinement of *F*^2^ against ALL reflections. The weighted *R*-factor *wR* and goodness of fit *S* are based on *F*^2^, conventional *R*-factors *R* are based on *F*, with *F* set to zero for negative *F*^2^. The threshold expression of *F*^2^ > σ(*F*^2^) is used only for calculating *R*-factors(gt) *etc*. and is not relevant to the choice of reflections for refinement. *R*-factors based on *F*^2^ are statistically about twice as large as those based on *F*, and *R*- factors based on ALL data will be even larger.

### Fractional atomic coordinates and isotropic or equivalent isotropic displacement parameters (Å^2^)

**Table:**

	*x*	*y*	*z*	*U*_iso_*/*U*_eq_	
Cl1	0.41647 (4)	0.38936 (6)	0.09436 (3)	0.07932 (19)	
Cl2	0.63726 (5)	0.49872 (9)	0.63868 (3)	0.1039 (3)	
O1	0.83676 (10)	0.43037 (13)	0.01202 (6)	0.0663 (4)	
O2	0.98385 (10)	0.45352 (13)	0.22616 (6)	0.0683 (4)	
H2	1.0287 (16)	0.411 (2)	0.2498 (11)	0.104 (9)*	
O3	0.81942 (10)	0.67287 (14)	−0.00658 (6)	0.0623 (3)	
H3	0.8184 (19)	0.5912 (17)	0.0018 (13)	0.101 (9)*	
O4	0.89094 (9)	0.81138 (12)	0.20647 (6)	0.0597 (3)	
O5	0.88325 (9)	0.38226 (15)	0.40367 (8)	0.0732 (4)	
H5	0.8578 (17)	0.4582 (18)	0.3958 (12)	0.088 (8)*	
O6	0.62092 (10)	0.14135 (13)	0.30146 (8)	0.0812 (5)	
O7	0.81303 (9)	0.60024 (13)	0.36945 (6)	0.0637 (3)	
O8	0.50411 (8)	0.49232 (11)	0.26293 (5)	0.0459 (3)	
H8	0.4609 (14)	0.5355 (19)	0.2384 (9)	0.073 (6)*	
C1	0.88850 (12)	0.40357 (16)	0.06380 (8)	0.0512 (4)	
C2	0.95946 (15)	0.29362 (19)	0.07285 (11)	0.0694 (5)	
H2A	1.0054	0.3029	0.0446	0.083*	
H2B	0.9266	0.2120	0.0641	0.083*	
C3	1.00944 (14)	0.30107 (18)	0.14210 (11)	0.0686 (5)	
H3A	1.0001	0.2226	0.1648	0.082*	
H3B	1.0777	0.3164	0.1455	0.082*	
C4	0.96129 (11)	0.41267 (15)	0.16725 (8)	0.0492 (4)	
C5	0.89209 (10)	0.46855 (14)	0.12298 (7)	0.0411 (3)	
C6	0.82777 (10)	0.57674 (13)	0.13687 (7)	0.0379 (3)	
H6A	0.8448	0.5903	0.1830	0.045*	
C7	0.84653 (9)	0.70428 (13)	0.10769 (7)	0.0379 (3)	
C8	0.87748 (10)	0.81257 (14)	0.14737 (8)	0.0429 (3)	
C9	0.89172 (12)	0.92755 (15)	0.10751 (9)	0.0524 (4)	
H9A	0.9571	0.9590	0.1183	0.063*	
H9B	0.8482	0.9964	0.1136	0.063*	
C10	0.87011 (12)	0.87857 (17)	0.03931 (9)	0.0533 (4)	
H10A	0.8178	0.9264	0.0141	0.064*	
H10B	0.9262	0.8852	0.0194	0.064*	
C11	0.84274 (10)	0.74123 (15)	0.04599 (7)	0.0446 (4)	
C12	0.72287 (10)	0.53369 (13)	0.12508 (6)	0.0382 (3)	
C13	0.65039 (11)	0.59634 (16)	0.08441 (8)	0.0490 (4)	
H13A	0.6651	0.6687	0.0625	0.059*	
C14	0.55591 (12)	0.55379 (19)	0.07543 (8)	0.0572 (4)	
H14A	0.5079	0.5975	0.0480	0.069*	
C15	0.53436 (12)	0.44653 (17)	0.10748 (8)	0.0523 (4)	
C16	0.60432 (13)	0.38306 (17)	0.14920 (9)	0.0565 (4)	
H16A	0.5890	0.3113	0.1713	0.068*	
C17	0.69758 (12)	0.42716 (15)	0.15791 (8)	0.0498 (4)	
H17A	0.7448	0.3847	0.1865	0.060*	
C18	0.82774 (11)	0.29123 (17)	0.37395 (8)	0.0518 (4)	
C19	0.87382 (14)	0.1676 (2)	0.36041 (11)	0.0701 (5)	
H19A	0.9061	0.1273	0.3995	0.084*	
H19B	0.9199	0.1813	0.3327	0.084*	
C20	0.79055 (16)	0.0862 (2)	0.32786 (12)	0.0773 (6)	
H20A	0.7991	0.0614	0.2855	0.093*	
H20B	0.7843	0.0091	0.3524	0.093*	
C21	0.70359 (12)	0.17179 (16)	0.32464 (8)	0.0531 (4)	
C22	0.73116 (10)	0.29280 (14)	0.35387 (7)	0.0410 (3)	
C23	0.65710 (10)	0.39155 (13)	0.36304 (7)	0.0372 (3)	
H23A	0.5950	0.3515	0.3464	0.045*	
C24	0.65913 (10)	0.51265 (14)	0.32455 (7)	0.0386 (3)	
C25	0.58503 (11)	0.55632 (14)	0.27997 (7)	0.0391 (3)	
C26	0.60407 (13)	0.68375 (15)	0.25230 (8)	0.0510 (4)	
H26A	0.5579	0.7477	0.2603	0.061*	
H26B	0.6020	0.6773	0.2067	0.061*	
C27	0.70464 (13)	0.71664 (16)	0.28730 (9)	0.0566 (4)	
H27A	0.7482	0.7258	0.2575	0.068*	
H27B	0.7043	0.7963	0.3109	0.068*	
C28	0.73447 (12)	0.60596 (15)	0.33194 (7)	0.0470 (4)	
C29	0.65562 (10)	0.42008 (14)	0.43328 (7)	0.0386 (3)	
C30	0.68823 (13)	0.33059 (18)	0.48066 (8)	0.0548 (4)	
H30A	0.7142	0.2534	0.4699	0.066*	
C31	0.68270 (14)	0.3545 (2)	0.54350 (9)	0.0666 (5)	
H31A	0.7051	0.2940	0.5748	0.080*	
C32	0.64416 (13)	0.4677 (2)	0.55949 (8)	0.0626 (5)	
C33	0.60995 (13)	0.55753 (19)	0.51394 (9)	0.0581 (4)	
H33A	0.5829	0.6337	0.5251	0.070*	
C34	0.61633 (11)	0.53321 (16)	0.45121 (8)	0.0465 (4)	
H34A	0.5937	0.5943	0.4203	0.056*	

### Atomic displacement parameters (Å^2^)

**Table:**

	*U*^11^	*U*^22^	*U*^33^	*U*^12^	*U*^13^	*U*^23^
Cl1	0.0615 (3)	0.0993 (4)	0.0808 (3)	−0.0363 (3)	0.0224 (2)	−0.0124 (3)
Cl2	0.1052 (5)	0.1636 (7)	0.0512 (3)	−0.0229 (4)	0.0355 (3)	−0.0156 (3)
O1	0.0814 (9)	0.0606 (8)	0.0530 (7)	0.0008 (7)	0.0025 (6)	−0.0173 (6)
O2	0.0733 (9)	0.0634 (8)	0.0587 (8)	0.0239 (7)	−0.0124 (6)	0.0061 (6)
O3	0.0812 (9)	0.0623 (8)	0.0424 (6)	−0.0042 (7)	0.0092 (6)	0.0008 (6)
O4	0.0650 (8)	0.0558 (7)	0.0537 (7)	−0.0155 (6)	−0.0012 (6)	−0.0125 (5)
O5	0.0374 (6)	0.0698 (9)	0.1046 (11)	−0.0064 (6)	−0.0072 (6)	−0.0178 (8)
O6	0.0644 (9)	0.0553 (8)	0.1104 (12)	−0.0106 (6)	−0.0193 (8)	−0.0238 (7)
O7	0.0544 (7)	0.0615 (8)	0.0717 (8)	−0.0214 (6)	0.0024 (6)	−0.0070 (6)
O8	0.0395 (6)	0.0471 (6)	0.0490 (6)	0.0049 (5)	0.0023 (5)	0.0088 (5)
C1	0.0516 (9)	0.0432 (9)	0.0596 (10)	−0.0035 (7)	0.0129 (8)	−0.0102 (7)
C2	0.0729 (12)	0.0506 (10)	0.0890 (14)	0.0098 (9)	0.0259 (11)	−0.0139 (10)
C3	0.0571 (11)	0.0494 (10)	0.0983 (15)	0.0155 (8)	0.0121 (10)	0.0000 (10)
C4	0.0442 (9)	0.0394 (8)	0.0625 (10)	0.0038 (6)	0.0058 (7)	0.0046 (7)
C5	0.0385 (8)	0.0353 (7)	0.0483 (8)	0.0001 (6)	0.0050 (6)	−0.0013 (6)
C6	0.0411 (8)	0.0365 (7)	0.0338 (7)	0.0031 (6)	0.0010 (6)	−0.0018 (5)
C7	0.0298 (7)	0.0378 (7)	0.0445 (8)	0.0019 (5)	0.0022 (6)	−0.0001 (6)
C8	0.0295 (7)	0.0422 (8)	0.0546 (9)	−0.0010 (6)	0.0012 (6)	−0.0043 (6)
C9	0.0392 (8)	0.0396 (8)	0.0776 (12)	−0.0026 (6)	0.0082 (8)	0.0013 (8)
C10	0.0436 (9)	0.0508 (9)	0.0670 (11)	0.0027 (7)	0.0143 (8)	0.0144 (8)
C11	0.0375 (8)	0.0477 (9)	0.0474 (8)	0.0021 (6)	0.0049 (6)	0.0032 (7)
C12	0.0447 (8)	0.0357 (7)	0.0346 (7)	0.0003 (6)	0.0089 (6)	−0.0042 (6)
C13	0.0450 (9)	0.0511 (9)	0.0483 (9)	−0.0078 (7)	0.0012 (7)	0.0091 (7)
C14	0.0456 (9)	0.0706 (12)	0.0522 (9)	−0.0086 (8)	0.0005 (7)	0.0054 (8)
C15	0.0518 (9)	0.0597 (10)	0.0489 (9)	−0.0163 (8)	0.0180 (7)	−0.0124 (8)
C16	0.0653 (11)	0.0472 (9)	0.0647 (11)	−0.0057 (8)	0.0318 (9)	0.0018 (8)
C17	0.0555 (10)	0.0447 (9)	0.0526 (9)	0.0072 (7)	0.0186 (7)	0.0076 (7)
C18	0.0378 (8)	0.0546 (10)	0.0612 (10)	−0.0008 (7)	0.0041 (7)	−0.0077 (8)
C19	0.0521 (10)	0.0676 (12)	0.0901 (14)	0.0150 (9)	0.0121 (10)	−0.0083 (10)
C20	0.0744 (13)	0.0565 (11)	0.0993 (16)	0.0124 (10)	0.0111 (11)	−0.0210 (11)
C21	0.0536 (10)	0.0442 (9)	0.0572 (10)	−0.0023 (7)	−0.0009 (8)	−0.0079 (7)
C22	0.0374 (7)	0.0404 (8)	0.0432 (8)	−0.0013 (6)	0.0025 (6)	−0.0035 (6)
C23	0.0304 (7)	0.0366 (7)	0.0421 (7)	−0.0042 (5)	0.0004 (5)	−0.0006 (6)
C24	0.0404 (8)	0.0386 (7)	0.0377 (7)	−0.0026 (6)	0.0094 (6)	−0.0027 (6)
C25	0.0454 (8)	0.0373 (7)	0.0372 (7)	0.0040 (6)	0.0146 (6)	−0.0011 (6)
C26	0.0673 (11)	0.0408 (8)	0.0491 (9)	0.0051 (7)	0.0216 (8)	0.0056 (7)
C27	0.0710 (11)	0.0411 (9)	0.0644 (10)	−0.0083 (8)	0.0297 (9)	−0.0014 (8)
C28	0.0512 (9)	0.0441 (8)	0.0484 (8)	−0.0093 (7)	0.0166 (7)	−0.0084 (7)
C29	0.0304 (7)	0.0425 (8)	0.0424 (7)	−0.0059 (6)	0.0056 (6)	0.0023 (6)
C30	0.0576 (10)	0.0560 (10)	0.0520 (9)	0.0051 (8)	0.0129 (8)	0.0113 (8)
C31	0.0658 (12)	0.0877 (15)	0.0475 (10)	−0.0002 (10)	0.0133 (8)	0.0200 (9)
C32	0.0511 (10)	0.0955 (15)	0.0449 (9)	−0.0167 (10)	0.0183 (8)	−0.0052 (9)
C33	0.0524 (10)	0.0649 (11)	0.0614 (11)	−0.0092 (8)	0.0219 (8)	−0.0140 (9)
C34	0.0428 (8)	0.0469 (9)	0.0506 (9)	−0.0018 (6)	0.0110 (7)	−0.0002 (7)

### Geometric parameters (Å, º)

**Table:**

Cl1—C15	1.7471 (17)	C13—H13A	0.9300
Cl2—C32	1.7414 (18)	C14—C15	1.372 (3)
O1—C1	1.240 (2)	C14—H14A	0.9300
O2—C4	1.310 (2)	C15—C16	1.374 (3)
O2—H2	0.858 (17)	C16—C17	1.379 (2)
O3—C11	1.318 (2)	C16—H16A	0.9300
O3—H3	0.869 (17)	C17—H17A	0.9300
O4—C8	1.2407 (19)	C18—C22	1.357 (2)
O5—C18	1.317 (2)	C18—C19	1.495 (3)
O5—H5	0.872 (16)	C19—C20	1.513 (3)
O6—C21	1.227 (2)	C19—H19A	0.9700
O7—C28	1.247 (2)	C19—H19B	0.9700
O8—C25	1.3182 (18)	C20—C21	1.512 (3)
O8—H8	0.856 (15)	C20—H20A	0.9700
C1—C5	1.425 (2)	C20—H20B	0.9700
C1—C2	1.512 (3)	C21—C22	1.427 (2)
C2—C3	1.519 (3)	C22—C23	1.507 (2)
C2—H2A	0.9700	C23—C24	1.507 (2)
C2—H2B	0.9700	C23—C29	1.532 (2)
C3—C4	1.495 (2)	C23—H23A	0.9800
C3—H3A	0.9700	C24—C25	1.359 (2)
C3—H3B	0.9700	C24—C28	1.430 (2)
C4—C5	1.361 (2)	C25—C26	1.496 (2)
C5—C6	1.512 (2)	C26—C27	1.522 (3)
C6—C7	1.510 (2)	C26—H26A	0.9700
C6—C12	1.529 (2)	C26—H26B	0.9700
C6—H6A	0.9800	C27—C28	1.504 (2)
C7—C11	1.364 (2)	C27—H27A	0.9700
C7—C8	1.429 (2)	C27—H27B	0.9700
C8—C9	1.503 (2)	C29—C34	1.386 (2)
C9—C10	1.520 (3)	C29—C30	1.389 (2)
C9—H9A	0.9700	C30—C31	1.381 (3)
C9—H9B	0.9700	C30—H30A	0.9300
C10—C11	1.494 (2)	C31—C32	1.367 (3)
C10—H10A	0.9700	C31—H31A	0.9300
C10—H10B	0.9700	C32—C33	1.371 (3)
C12—C13	1.380 (2)	C33—C34	1.382 (2)
C12—C17	1.393 (2)	C33—H33A	0.9300
C13—C14	1.390 (2)	C34—H34A	0.9300
			
C4—O2—H2	114.9 (18)	C16—C17—H17A	119.1
C11—O3—H3	111.2 (18)	C12—C17—H17A	119.1
C18—O5—H5	111.7 (17)	O5—C18—C22	128.46 (16)
C25—O8—H8	113.0 (15)	O5—C18—C19	117.91 (15)
O1—C1—C5	127.01 (15)	C22—C18—C19	113.62 (15)
O1—C1—C2	123.65 (16)	C18—C19—C20	103.78 (15)
C5—C1—C2	109.34 (15)	C18—C19—H19A	111.0
C1—C2—C3	105.41 (15)	C20—C19—H19A	111.0
C1—C2—H2A	110.7	C18—C19—H19B	111.0
C3—C2—H2A	110.7	C20—C19—H19B	111.0
C1—C2—H2B	110.7	H19A—C19—H19B	109.0
C3—C2—H2B	110.7	C21—C20—C19	104.60 (15)
H2A—C2—H2B	108.8	C21—C20—H20A	110.8
C4—C3—C2	103.13 (15)	C19—C20—H20A	110.8
C4—C3—H3A	111.1	C21—C20—H20B	110.8
C2—C3—H3A	111.1	C19—C20—H20B	110.8
C4—C3—H3B	111.1	H20A—C20—H20B	108.9
C2—C3—H3B	111.1	O6—C21—C22	124.64 (16)
H3A—C3—H3B	109.1	O6—C21—C20	125.22 (16)
O2—C4—C5	123.05 (15)	C22—C21—C20	110.13 (14)
O2—C4—C3	123.33 (15)	C18—C22—C21	107.86 (14)
C5—C4—C3	113.62 (16)	C18—C22—C23	130.81 (14)
C4—C5—C1	108.45 (14)	C21—C22—C23	121.10 (13)
C4—C5—C6	124.43 (14)	C24—C23—C22	114.51 (12)
C1—C5—C6	127.05 (13)	C24—C23—C29	112.14 (12)
C7—C6—C5	114.49 (12)	C22—C23—C29	113.24 (11)
C7—C6—C12	115.42 (11)	C24—C23—H23A	105.3
C5—C6—C12	110.69 (11)	C22—C23—H23A	105.3
C7—C6—H6A	105.0	C29—C23—H23A	105.3
C5—C6—H6A	105.0	C25—C24—C28	108.40 (14)
C12—C6—H6A	105.0	C25—C24—C23	124.83 (13)
C11—C7—C8	107.68 (13)	C28—C24—C23	126.57 (13)
C11—C7—C6	131.89 (13)	O8—C25—C24	123.07 (13)
C8—C7—C6	120.40 (13)	O8—C25—C26	123.34 (13)
O4—C8—C7	124.60 (14)	C24—C25—C26	113.59 (14)
O4—C8—C9	124.90 (14)	C25—C26—C27	103.07 (13)
C7—C8—C9	110.50 (13)	C25—C26—H26A	111.1
C8—C9—C10	104.56 (13)	C27—C26—H26A	111.1
C8—C9—H9A	110.8	C25—C26—H26B	111.1
C10—C9—H9A	110.8	C27—C26—H26B	111.1
C8—C9—H9B	110.8	H26A—C26—H26B	109.1
C10—C9—H9B	110.8	C28—C27—C26	105.52 (13)
H9A—C9—H9B	108.9	C28—C27—H27A	110.6
C11—C10—C9	103.83 (13)	C26—C27—H27A	110.6
C11—C10—H10A	111.0	C28—C27—H27B	110.6
C9—C10—H10A	111.0	C26—C27—H27B	110.6
C11—C10—H10B	111.0	H27A—C27—H27B	108.8
C9—C10—H10B	111.0	O7—C28—C24	126.78 (15)
H10A—C10—H10B	109.0	O7—C28—C27	123.81 (15)
O3—C11—C7	129.01 (15)	C24—C28—C27	109.40 (14)
O3—C11—C10	117.60 (14)	C34—C29—C30	117.67 (15)
C7—C11—C10	113.39 (14)	C34—C29—C23	120.89 (13)
C13—C12—C17	117.47 (14)	C30—C29—C23	121.32 (14)
C13—C12—C6	123.65 (13)	C31—C30—C29	121.05 (17)
C17—C12—C6	118.87 (13)	C31—C30—H30A	119.5
C12—C13—C14	121.54 (15)	C29—C30—H30A	119.5
C12—C13—H13A	119.2	C32—C31—C30	119.64 (17)
C14—C13—H13A	119.2	C32—C31—H31A	120.2
C15—C14—C13	119.18 (16)	C30—C31—H31A	120.2
C15—C14—H14A	120.4	C31—C32—C33	121.00 (17)
C13—C14—H14A	120.4	C31—C32—Cl2	119.89 (16)
C14—C15—C16	120.89 (16)	C33—C32—Cl2	119.11 (17)
C14—C15—Cl1	119.41 (14)	C32—C33—C34	119.00 (18)
C16—C15—Cl1	119.70 (14)	C32—C33—H33A	120.5
C15—C16—C17	119.17 (16)	C34—C33—H33A	120.5
C15—C16—H16A	120.4	C33—C34—C29	121.64 (16)
C17—C16—H16A	120.4	C33—C34—H34A	119.2
C16—C17—C12	121.72 (16)	C29—C34—H34A	119.2
			
O1—C1—C2—C3	177.35 (18)	O5—C18—C19—C20	178.89 (19)
C5—C1—C2—C3	−2.3 (2)	C22—C18—C19—C20	−0.2 (2)
C1—C2—C3—C4	1.3 (2)	C18—C19—C20—C21	0.9 (2)
C2—C3—C4—O2	−179.42 (17)	C19—C20—C21—O6	−179.9 (2)
C2—C3—C4—C5	0.2 (2)	C19—C20—C21—C22	−1.3 (2)
O2—C4—C5—C1	177.93 (16)	O5—C18—C22—C21	−179.57 (19)
C3—C4—C5—C1	−1.6 (2)	C19—C18—C22—C21	−0.5 (2)
O2—C4—C5—C6	−4.9 (3)	O5—C18—C22—C23	−5.2 (3)
C3—C4—C5—C6	175.49 (15)	C19—C18—C22—C23	173.81 (17)
O1—C1—C5—C4	−177.19 (17)	O6—C21—C22—C18	179.83 (19)
C2—C1—C5—C4	2.5 (2)	C20—C21—C22—C18	1.1 (2)
O1—C1—C5—C6	5.8 (3)	O6—C21—C22—C23	4.8 (3)
C2—C1—C5—C6	−174.57 (15)	C20—C21—C22—C23	−173.88 (16)
C4—C5—C6—C7	110.93 (17)	C18—C22—C23—C24	72.4 (2)
C1—C5—C6—C7	−72.47 (19)	C21—C22—C23—C24	−113.89 (16)
C4—C5—C6—C12	−116.52 (16)	C18—C22—C23—C29	−57.9 (2)
C1—C5—C6—C12	60.1 (2)	C21—C22—C23—C29	115.82 (15)
C5—C6—C7—C11	60.0 (2)	C22—C23—C24—C25	119.43 (15)
C12—C6—C7—C11	−70.2 (2)	C29—C23—C24—C25	−109.75 (15)
C5—C6—C7—C8	−117.54 (14)	C22—C23—C24—C28	−66.24 (18)
C12—C6—C7—C8	112.20 (14)	C29—C23—C24—C28	64.58 (18)
C11—C7—C8—O4	−178.14 (14)	C28—C24—C25—O8	−179.23 (13)
C6—C7—C8—O4	−0.1 (2)	C23—C24—C25—O8	−4.0 (2)
C11—C7—C8—C9	1.64 (17)	C28—C24—C25—C26	0.67 (17)
C6—C7—C8—C9	179.73 (12)	C23—C24—C25—C26	175.88 (13)
O4—C8—C9—C10	177.76 (14)	O8—C25—C26—C27	179.57 (14)
C7—C8—C9—C10	−2.02 (17)	C24—C25—C26—C27	−0.33 (17)
C8—C9—C10—C11	1.57 (16)	C25—C26—C27—C28	−0.13 (16)
C8—C7—C11—O3	178.88 (15)	C25—C24—C28—O7	178.60 (16)
C6—C7—C11—O3	1.1 (3)	C23—C24—C28—O7	3.5 (3)
C8—C7—C11—C10	−0.56 (17)	C25—C24—C28—C27	−0.74 (17)
C6—C7—C11—C10	−178.35 (14)	C23—C24—C28—C27	−175.84 (14)
C9—C10—C11—O3	179.79 (14)	C26—C27—C28—O7	−178.84 (15)
C9—C10—C11—C7	−0.69 (18)	C26—C27—C28—C24	0.52 (17)
C7—C6—C12—C13	7.8 (2)	C24—C23—C29—C34	27.75 (18)
C5—C6—C12—C13	−124.24 (16)	C22—C23—C29—C34	159.21 (13)
C7—C6—C12—C17	−170.70 (13)	C24—C23—C29—C30	−156.14 (14)
C5—C6—C12—C17	57.23 (17)	C22—C23—C29—C30	−24.67 (19)
C17—C12—C13—C14	−1.2 (2)	C34—C29—C30—C31	−0.8 (2)
C6—C12—C13—C14	−179.74 (15)	C23—C29—C30—C31	−177.04 (15)
C12—C13—C14—C15	−0.4 (3)	C29—C30—C31—C32	0.3 (3)
C13—C14—C15—C16	1.6 (3)	C30—C31—C32—C33	0.7 (3)
C13—C14—C15—Cl1	−177.84 (14)	C30—C31—C32—Cl2	−179.82 (14)
C14—C15—C16—C17	−1.1 (3)	C31—C32—C33—C34	−1.1 (3)
Cl1—C15—C16—C17	178.33 (13)	Cl2—C32—C33—C34	179.40 (13)
C15—C16—C17—C12	−0.6 (3)	C32—C33—C34—C29	0.5 (2)
C13—C12—C17—C16	1.7 (2)	C30—C29—C34—C33	0.4 (2)
C6—C12—C17—C16	−179.68 (14)	C23—C29—C34—C33	176.63 (14)

### Hydrogen-bond geometry (Å, º)

**Table:**

*D*—H···*A*	*D*—H	H···*A*	*D*···*A*	*D*—H···*A*
O5—H5···O7	0.87 (2)	1.66 (2)	2.529 (2)	171 (3)
O3—H3···O1	0.87 (2)	1.70 (2)	2.558 (2)	169 (3)
O2—H2···O4^i^	0.86 (2)	1.69 (2)	2.5424 (16)	173 (3)
O8—H8···O6^ii^	0.86 (2)	1.71 (2)	2.5564 (16)	171 (2)
C9—H9*A*···O5^iii^	0.97	2.52	3.277 (2)	134

Symmetry codes: (i) −*x*+2, *y*−1/2, −*z*+1/2; (ii) −*x*+1, *y*+1/2, −*z*+1/2; (iii) −*x*+2, *y*+1/2, −*z*+1/2.
